# Impact of PEDV infection on the biological characteristics of porcine intestinal exosomes

**DOI:** 10.3389/fmicb.2024.1392450

**Published:** 2024-05-13

**Authors:** Junjie Wu, Langju Su, Guangmiao Ma, Yichen Wang, Yuhang Luo, Saeed EI-Ashram, Reem Atalla Alajmi, Zhili Li

**Affiliations:** ^1^College of Life Science and Engineering, Foshan University, Foshan, China; ^2^Department of Zoology, Faculty of Science, Kafrelsheikh University, Kafr EI-Sheikh, Egypt; ^3^Department of Zoology, Faculty of Science, King Saud University, Riyadh, Saudi Arabia

**Keywords:** exosomes, microRNA, ultracentrifugation, porcine intestinal tissue damage, porcine epidemic diarrhea virus

## Abstract

Porcine epidemic diarrhea (PED) is a highly contagious intestinal infection primarily affecting pigs. It is caused by the porcine epidemic diarrhea virus (PEDV). PEDV targets the villus tissue cells in the small intestine and mesenteric lymph nodes, resulting in shortened intestinal villi and, in extreme cases, causing necrosis of the intestinal lining. Moreover, PEDV infection can disrupt the balance of the intestinal microflora, leading to an overgrowth of harmful bacteria like *Escherichia coli*. Exosomes, tiny membrane vesicles ranging from 30 to 150 nm in size, contain a complex mixture of RNA and proteins. MicroRNA (miRNA) regulates various cell signaling, development, and disease progression processes. This study extracted exosomes from both groups and performed high-throughput miRNA sequencing and bioinformatics techniques to investigate differences in miRNA expression within exosomes isolated from PEDV-infected porcine small intestine tissue compared to healthy controls. Notably, two miRNA types displayed upregulation in infected exosomes, while 12 exhibited downregulation. These findings unveil abnormal miRNA regulation patterns in PEDV-infected intestinal exosomes, shedding light on the intricate interplay between PEDV and its host. This will enable further exploration of the relationship between these miRNA changes and signaling pathways, enlightening PEDV pathogenesis and potential therapeutic targets.

## 1 Introduction

Porcine epidemic diarrhea (PED), a highly infectious intestinal disease affecting pigs, is caused by the porcine epidemic diarrhea virus (PEDV). This widespread and potentially devastating disease manifests in afflicted animals through symptoms like diarrhea, vomiting, and dehydration. PEDV's classification as a coronavirus, specifically within the genus coronavirus and coronaviridae family, underscores its kinship with other coronaviruses, including those known to infect humans. This shared lineage highlights the importance of understanding and controlling PEDV outbreaks (Song et al., 2015).

PEDV closely resembles the morphology and structure of other coronaviruses, showcasing typical characteristics of the coronavirus family. Viral particles found in fecal samples typically exhibit a spherical shape and range in size from 95 to 190 nm, with an average diameter of ~130 nm, including surface projections. Radially arranged rod-shaped fibroids, measuring 18–23 nm, comprise the capsule layer enveloping the virus. At the core of most PEDV virions is an electronically dense region, which can be challenging to distinguish morphologically from the Porcine Transmissible Gastroenteritis Virus (TGEV).

Like other coronaviruses, PEDV replicates within intestinal tissue cells' cytoplasm and assembles through a budding process via the endoplasmic reticulum membrane. This replication mechanism highlights the similarities among coronaviruses and provides a foundation for understanding and combating PEDV infections.

Piglets are the most susceptible group to PEDs, but all age categories can become infected. The disease particularly affects nursery, grower, and finishing pigs, with the most severely impacted nursing piglets. The incidence of infection among sows can range from 15 to 90%. The primary source of infection is typically sick pigs. PEDV is known to be present in intestinal villi tissue cells and mesenteric lymph nodes, and it spreads through contaminated feces in the environment, feed, water, transportation, and utensils. The digestive tract is the primary entry point for PEDV infection. If a farm experiences frequent piglet litters, the virus can persist and infect weaned piglets that have lost maternal antibodies, leading to endemic PED.

Histological examination of infected piglets' intestinal tissue reveals significant atrophy of the small intestine's villi, a decreased ratio of intestinal villi height to crypt depth in the jejunum, ileum, and cecum, as well as congestion in the lamina propria, as observed through a microscope. These findings highlight the severity of intestinal tissue damage caused by PEDs, emphasizing the importance of disease prevention and control strategies (Jung and Saif, 2017).

Infection of piglets with PEDV resulted in swelling of the small intestine and hyaline intestinal wall tissue. HE staining of jejunal sections showed damaged and lysed small intestinal villi, vacuolated cytoplasm, and degeneration of mucosal epithelial cells, accompanied by a slight lymphocytic infiltration. Immunohistochemical staining clearly showed that the epithelium of the jejunum contained antigens, and the intestinal glands of the cecum also had a small amount of antigens (Li et al., 2022). Minimal pathological changes are observed in the large intestine, although a reduction in cuprocyte numbers is noticeable. Moreover, PEDV infection has been found to significantly disrupt the colonization of newborn piglets' intestinal microflora. The number of beneficial bacteria, such as *Bacteroides*, decreases significantly, as does the population of *Firmicutes*, which includes pathogenic members of the Bacillus and Clostridium genera (e.g., Clostridium perfringens, Clostridium spp.). Additionally, Proteobacteria, primarily representing pathogenic bacteria within the *Enterobacteriaceae* family (e.g., *Escherichia, Salmonella, Shigella*, and *Clostridium* genera), show a considerable increase. This imbalance in intestinal flora can exacerbate PEDV infection. Among the various pathogenic bacteria, *Clostridium perfringens* and *Clostridium* spp. are particularly noteworthy. These findings emphasize the importance of maintaining a balanced intestinal microflora to mitigate the severity of PEDV infections in piglets. Compared to healthy individuals, the number of Clostridium perfringens in infected sows and piglets increases by 10–15. In most cases, Clostridium perfringens type C, as the primary pathogen of the intestinal tract, infects the intestinal epithelium as a secondary infection following PED, infectious gastroenteritis, or rotavirus infections, worsening the disease. Under normal circumstances, *Clostridium perfringens* type C is rarely found in the intestines of healthy pigs. The infectivity remains unaltered despite ultrasonic treatment or repeated freeze-thaw cycles. The virus contains RNA as its nucleic acid, and 5-iodine-2-deoxyuridine does not impact its replication. Research suggests that PEDV-infected piglets can influence exosome production in porcine intestinal tissue, affecting the tissue's replication process. miRNA, a single-stranded RNA molecule with regulatory functions widely present *in vivo*, is also an essential component of exosomes. Limited studies have investigated exosomes and their internal miRNAs derived from PEDV-infected pig small intestine tissue. Identifying miRNAs in exosomes from the small intestine of PEDV-infected pigs and analyzing the differential expression of abnormally regulated miRNAs through sequencing analysis and bioinformatics may contribute to a better understanding the interaction between PEDV and its host. These insights could lead to the development of improved disease management strategies.

Exosomes, or intracavicular vesicles (ILVs), are small membrane vesicles (30–150 nm) containing complex RNAs and proteins. They are secreted by all cell types and can be found in body fluids (Hornick et al., 2015). Exosomes are extracellular nanovesicles formed through the “endocytosis-fusion-efflorescence” process, carrying nucleic acids (such as microRNA and mRNA), proteins, lipids, and other bioactive molecules into recipient cells, thereby regulating their phenotype and function. Exosomes, therefore, play a crucial role in cell-cell and tissue-tissue information exchange and regulation. Intestinal tissue has the largest surface area among organs, consisting of a single layer of cells. Consequently, we decided to extract exosomes from porcine small intestine tissue for our study. MicroRNA (miRNA) is a non-coding short-sequence RNA (22–23 nucleotides) found in both eukaryotes and viruses. The interspecific evolutionary sequence is highly conserved, and miRNAs' timing and tissue specificity are clear (Correia de Sousa et al., 2019). Although over 5,500 miRNAs have been predicted, only a few have been confirmed for their biological functions. Nevertheless, the range of miRNAs' action is extremely broad. As revealed by bioinformatics software analysis, miRNAs can target ~30% of the mRNA in the body, regulating various life activities (Londin et al., 2015).

Through regulation of gene expression during various stages of life processes, including cell growth, tissue differentiation, embryonic development, body growth, disease onset, and immune regulation in organisms, miRNAs play crucial roles in living beings (Correia de Sousa et al., 2019). In addition to their role within cells, miRNAs also play diverse regulatory roles as vital components of exosomes, which serve as promising vehicles for delivering therapeutic molecules like interfering RNA and other substances. Researchers have increasingly focused on the regulatory role of miRNA in the intricate relationship between pathogen and host. This study aimed to investigate the specific miRNAs involved in this interaction by isolating exosomes from both PEDV-infected and healthy porcine small intestine tissues. High-throughput sequencing was utilized to identify miRNAs within the exosomes, followed by sequencing analysis and bioinformatics techniques to compare and analyze miRNA expression differences in the exosomes derived from PEDV-infected porcine small intestine tissues.

## 2 Materials and methods

### 2.1 Grouping and handling of test animals

Six healthy newborn piglets, all with negative PEDV antigen and antibody levels, were sourced from a pig farm in Guangxi Province. The piglets were assigned to either the PEDV-infected or the control group, with three piglets in each group. At the age of 3 days, the piglets were weaned and transferred to the laboratory animal housing for artificial feeding without receiving any colostrum or antibiotics. The piglets in the PEDV-infected group were orally administered 2 ml of PEDV at a concentration of 10^3^ TCID_50_, while the control group received an equal volume of PBS. The jejunum, ileum, and cecum tissues from the PEDV-infected group were collected when all piglets succumbed to the disease. The tissues were rinsed with PBS, frozen in liquid nitrogen, and stored for further analysis. The PEDV JS-2013 strain used in the study was generously provided by the Shanghai Institute of Veterinary Medicine, Chinese Academy of Agricultural Sciences.

### 2.2 Characterization and isolation of exosomes from swine intestinal tissue

Porcine small intestinal tissue samples were collected from both PEDV-infected and healthy newborn piglets. After rinsing the tissues with ice-cold PBS to remove intestinal contents and mucosa, they were cut into ~0.4 cm-long pieces and immersed in HBSS buffer solution containing type I collagen (300 units/ml) at 37°C for 30 min. The mixture was placed on ice to halt enzyme digestion, and an equal volume of PBS and 2 × protease inhibitor cocktail was added. The homogenized tissue samples were filtered through a 40-micron sterile nylon mesh filter into new tubes. The filtrate was centrifuged at 1,000 × g for 10 min to remove cell fragments, and the supernatant was transferred to a new 15 ml tube. The supernatant was centrifuged at 2,000 × g for 20 min, 5,000 × g for 30 min, and 15,000 × g for 1 h at 4°C to remove cell debris. Next, the supernatant was filtered using a 0.22 μm Millex PVDF filter into new 15 ml tubes. The purified liquid was transferred into a new 5 ml tube, and ExojuiceTM (100,000 × g) was added for 70 min at room temperature. The initial 250 μl of liquid at the bottom of the tube was discarded, and 150 μl of liquid containing purified exosomes was collected and stored at −80°C for later use. The exosomes were dialyzed to remove the ExojuiceTM reagent by placing them in a dialysis bag (MWCO1KD) and incubating in 1 × PBS buffer solution for 3 h at 4°C. The suspension of exosomes on the membrane was collected and stored at −80°C for later use.

### 2.3 Western blot analysis of exosomal marker protein

The exosome pellets were resuspended in PBS and combined with RIPA lysis buffer to extract proteins. The protein lysate was mixed with 5 × loading buffers and boiled at 98°C for 7 min. The protein samples were then separated using a 12% SDS-PAGE gel and transferred to a PVDF membrane. The membrane was immersed in purified methanol for 15 s, followed by deionized water for 2 min. The membrane was activated in a filter-membrane-rubber-filter transfer solution for 20 min. The filter-membrane-rubber filter was placed on the semi-dry transfer device, and the proteins were transferred for 20 min at 20 volts. The PVDF membrane was blocked in 5% skim milk powder and TBST for 1 h at room temperature on a shaker. After removing the blocking solution, the membrane was washed with PBST. The primary antibodies were diluted with TBST and incubated overnight at 4°C on a constant temperature shaker. The next day, the membrane was washed with TBST and incubated with the secondary antibody for 2 h at room temperature. Afterwards, the membrane was washed with TBST and stained with a BCIP/NBT chromogenic substrate kit. The results were recorded and analyzed.

### 2.4 Transmission electron microscopy

Exosome morphology was examined using transmission electron microscopy (TEM). The exosome suspension was combined with a 0.2% paraformaldehyde solution and placed on a carbon-coated copper grid. After staining with 1% uranyl acetate for 2 min, the grid samples were filtered and dried for 10 min. A transmission electron microscope (JEM-1400, Japan) was used to observe the grid samples.

### 2.5 Nanoparticle tracking analysis

Nanoparticle tracking analysis (NTA) was used to analyze exosome concentration and size distribution. Particles were automatically tracked and identified based on Brownian motion and diffusion coefficient. To evaluate the concentration and particle size, the sample pool was cleaned with deionized water, and the isolated exosome suspension was diluted 4,000 times with 1 × PBS buffer.

### 2.6 Cytotoxicity analysis of extracellular vesicles

#### 2.6.1 Cell counting kit-8 assay

Cell viability was measured using the cell counting kit-8 (CCK-8, Monmouth Junction, NY, USA). Vero cells (5 × 10^5^ cells/well) were cultivated in 96-well plates for 36 h at 37°C. The cells were washed three times with PBS, then different concentrations of exosomes (0, 0.1, 1, 10, 50, 100, and 1,000 μg/ml) were added to the corresponding well. After 1 h cultivation, 10 μl CCK-8 reagent was added to each well and the cells were incubated at 37°C for 2 h. The OD value was detected at 450 nm using a microplatereader (BioTex TX800, USA).

#### 2.6.2 PEDV titration

The virus titer was measured using the tissue culture infectious dose 50% (TCID50) test. Vero cells were seeded into 96-well plates at a density of 5 × 10^5^ cells per well and cultivated for 36 h at 37°C. Before infection, the media was withdrawn, and the cells were washed twice with PBS before adding 0.2 ml of 10-fold serial virus dilutions in DMEM to each well. For 5 days, the CPE was examined under a microscope, and the cytopathology was monitored every 12 h. Then, the Reed and Munch technique was used to compute the TCID50.

#### 2.6.3 Co-incubation

Vero cells were grown in DMEM (Gibco, USA) with 10% FBS (Gibco, USA) and 1% penicillin-streptomycin in 24-well plates to evaluate the activity of two kinds of exosomes. After 36 h, the cells were 90% confluent, washed three times with PBS, and 0, 0.05, 0.5, 5, 2.5, and 50 μg of exosomes were introduced to the appropriate well and incubated at 37°C for 1 h. The cells were washed three times with PBS, inoculated with the PEDV at MOI = 0.001, and incubated at 37°C for 1.5 h. The cells were grown in 500 μl of complete culture media after a second PBS wash. At 24 h post-infection, three cycles of freezing and thawing were conducted, and the supernatants were collected for examination of virus proliferation by cytopathic effect, PEDV gene copy number, TCID50 test, and production of PEDV nucleocapsid protein in Vero cells.

### 2.7 Identification of differentially expressed exosomal miRNAs

#### 2.7.1 PEDV-infected and healthy pig small intestine exosome RNA extraction

RNA was extracted and purified from PEDV-infected and healthy porcine intestinal exosomes using the miRNeasy Mini kit, following the manufacturer's instructions.

#### 2.7.2 Library construction and sequencing

The RNA sample quality was assessed using the Agilent 2100 Pic600. Once qualified, the library was constructed using the Small RNA Sample Prep Kit. Reverse transcription was performed on total RNA, followed by PCR and cDNA recovery to create the sequencing library. The library's consistency was evaluated using the Agilent 2100 and qPCR. Following the assessment, Illumina SE50 sequencing was performed on the qualified library.

#### 2.7.3 Sequencing data quality control and filtering

Quality control of the raw data was conducted to ensure the accuracy of the information analysis. Raw reads from sequencing were processed and filtered to obtain high-quality sequences (clean reads). Reads that lacked 3′ and 5′ joint sequences, inserted fragments, N bases >10%, and continuous A/T/G/C bases were excluded from the original data. The resulting data contained clean reads for further analysis.

#### 2.7.4 miRNA identification and prediction

Sequences between 18 and 35 nt in length were selected for analysis, corresponding to the typical miRNA size. After Bowtie mapped the sRNAs to the Sus scrofa 11.1 genome, the expression and distribution of small RNA on the reference sequence were examined. The mature pig miRNA sequences in the miRBase database (V22) were compared with the sRNA sequences matched on the reference genome to identify the details of the matched miRNAs of each sample, including the secondary structure of the known matched miRNAs. The length, frequency, and sequence of miRNAs within each sample were determined. Finally, based on the unique hairpin structure of miRNA precursors, miREvo software was used to predict potential new miRNAs from unmatched sequences.

#### 2.7.5 Analysis of differential expression of miRNA

The expression levels of known and novel miRNAs were determined in each sample and normalized to TPM. Normalized expression was calculated using the formula: normalized expression = mapped read count/total read count of compared miRNAs ^*^ 1,000,000. Low-expressed miRNAs (TPM <10) were excluded to ensure high-quality variance analysis results. To control the false positive rate between repeated samples, the difference in miRNA expression analysis was set to |log_2_ (foldchange)| ≥ 1 and *P*-value ≤ 0.05, and these were designated as differentially expressed. Hierarchical clustering analysis of all differentially expressed miRNAs was conducted using the pheatmap R package.

#### 2.7.6 Target gene prediction and enrichment analysis

To predict miRNA target genes, MiRanda and RNAhybrid were employed. For functional annotation and pathway analysis, the GO (geneontology.org) and KEGG (genome.jp/kegg/) databases were utilized.

### 2.8 RT-qPCR to verify miRNA expression

#### 2.8.1 Design and synthesis of primers

Eight differentially expressed miRNAs were randomly selected from the miRBase database (V22) to identify miRNA sequences. Primer sequences were designed for miRNA expression confirmation using the Primer 5 program, as shown in Table 1.

**Table 1:** Primer sequences of miRNA.

**miRNA name**	**Primers sequence (5^′^-3^′^)**
ssc-miR-486	TCCTGTACTGAGCTGCCCCGA
ssc-miR-215	GCGCATGACCTATGAATTGACAGAC
ssc-miR-363	CGGAATTGCACGGTATCCATCTGTAA
ssc-miR-142-5p	GCGGCCATAAAGTAGAAAGCACTACT
ssc-miR-122-5p	GTGGAGTGTGACAATGGTGTTTGT
ssc-miR-451	GGCGAAACCGTTACCATTACTGAGTT
ssc-miR-132	GGCGTAACAGTCTACAGCCATGGTCG
ssc-miR-181d-5p	AACATTCATTGTTGTCGGTGGGTT

#### 2.8.2 Reverse transcription

The reaction system was prepared according to the miRcute Plus miRNA First-Strand cDNA Kit instructions, and the procedure was carried out. The reaction conditions were: 42°C for 60 min and 95°C for 3 min. The reaction system is displayed in Table 2.

**Table 2:** Reverse transcription system of miRNA.

**Reagent**	**Volume/μL**
Total RNA	1
2 × miRNA RT reaction buffer	10
mRNA RT enzyme mix	2
RNase-free ddH_2_O	7
Total volume	20
Total RNA	1

## 3 Results

### 3.1 Characteristic lesions of the intestinal tract in piglets

#### 3.1.1 Autopsy lesions

**Figure 1:**
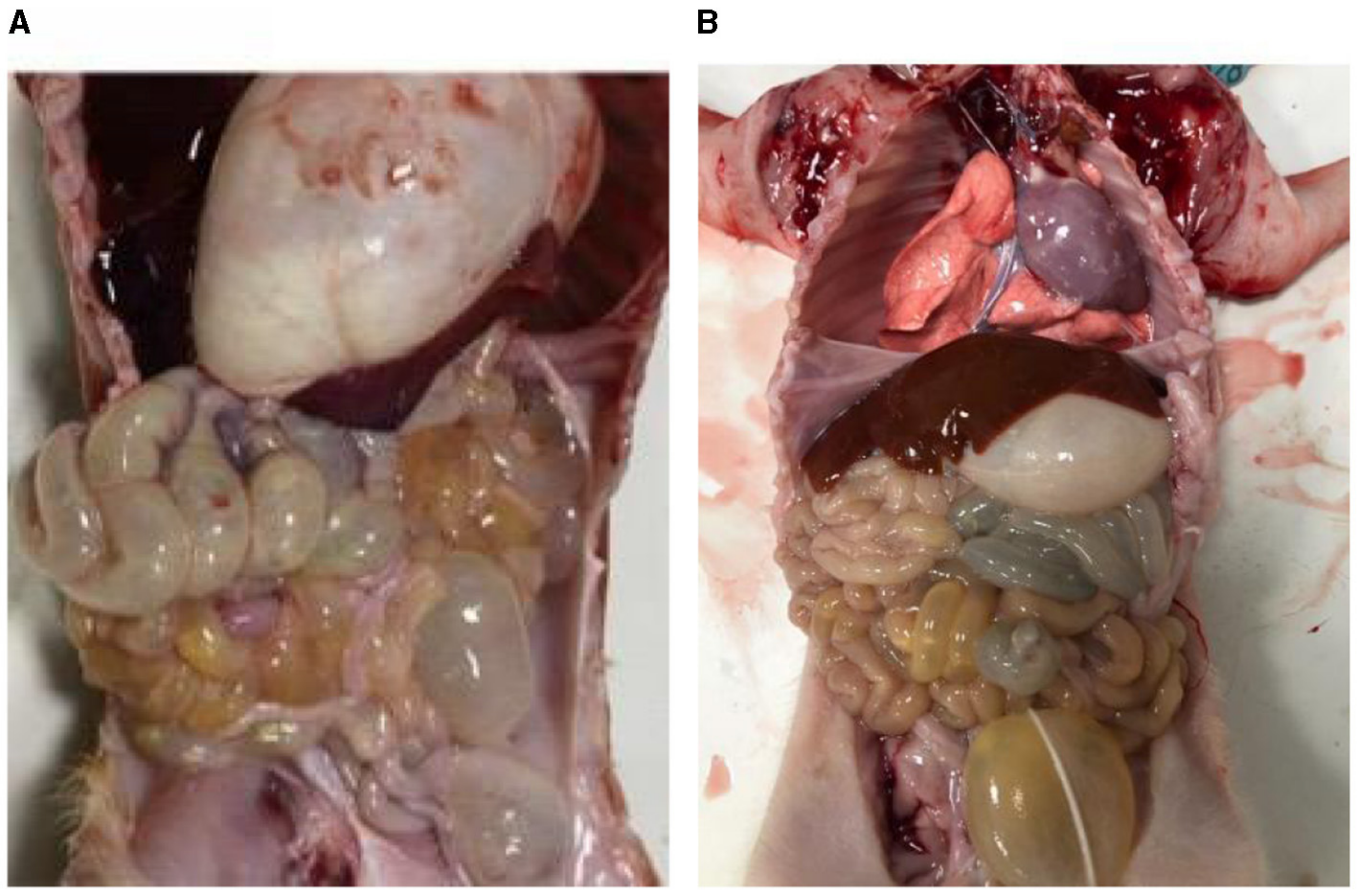
Piglet autopsy lesions. **(A)** Anatomy of the PEDV-infected group. **(B)** Anatomy of healthy piglets.

The small intestines of the infected piglets (Figure 1A) appeared partially swollen and transparent compared to those of the control piglets (Figure 1B). Additionally, fluid was observed in the intestinal lumen, and undigested milky-white chyme was present in the stomach of the infected piglets.

### 3.2 Analysis of exosome characteristics

**Figure 2:**
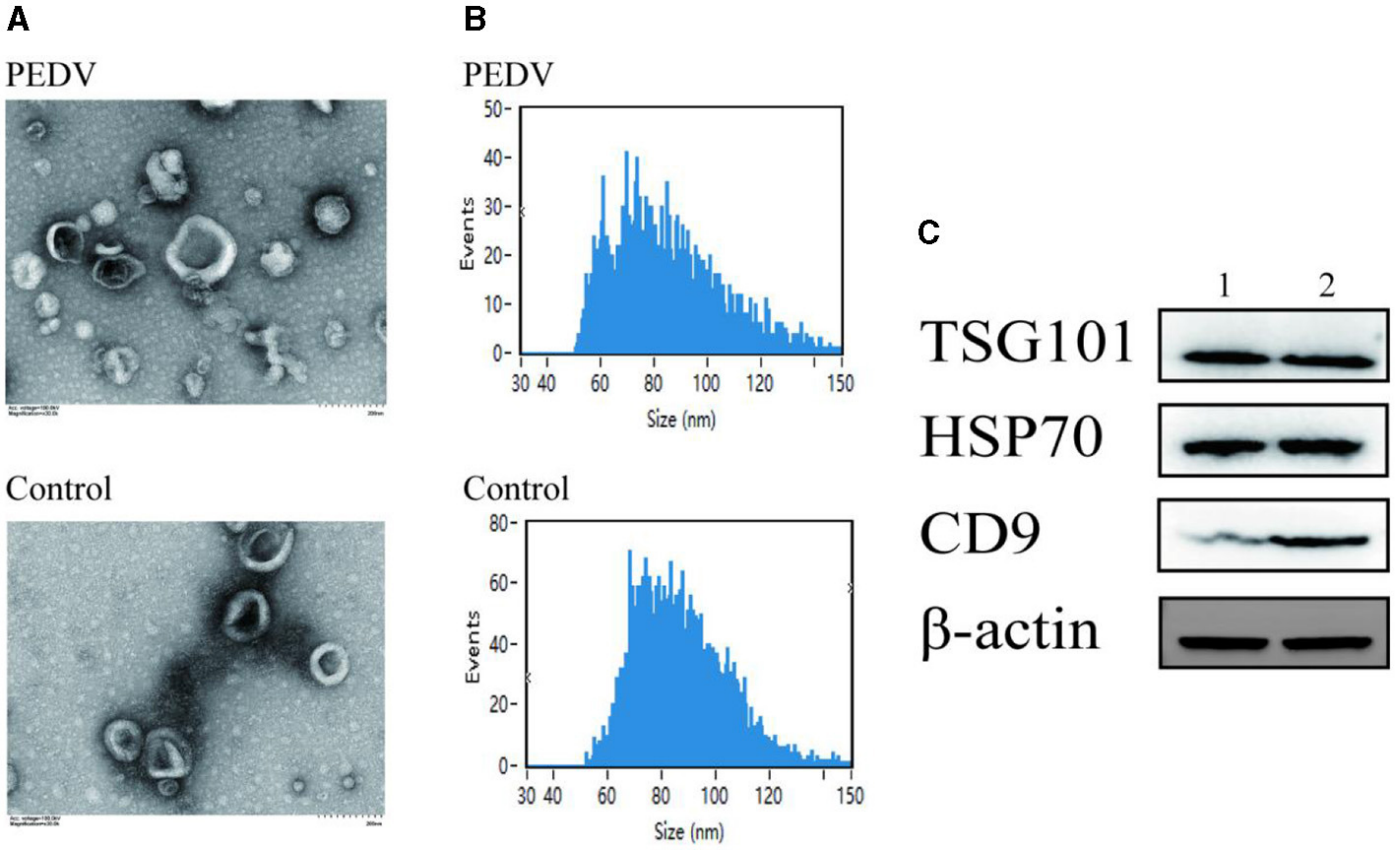
Porcine intestine exosome identification. **(A)** Photomicrographs of exosomes acquired using transmission electron microscopy. **(B)** NanoFCM study of exosome particle size. **(C)** Western blotting was utilized to identify the expression of biomarker proteins, such as TSG101, HSP70, and CD9. Lane 1, Healthy piglets' intestinal tissue exosome. Lane 2, PEDV-infected piglets' intestinal tissue exosomes.

Transmission electron microscopy was used to examine the size of exosomes in small intestinal tissues of piglets from the PEDV-infected group and the control group (Figure 2A). The results demonstrated that exosomes appeared as circular vesicle structures with a diameter of 30–200 nm, and PEDV infection did not affect the size of exosomes in piglet small intestinal tissues. Nanoparticle tracking analysis revealed that the particle size of exosomes in porcine small intestine tissue ranged from 50 to 150 nm (Figure 2B). The number of exosomes in pig small intestine tissue with particle sizes ranging from 50 to 70 nm was higher in the PEDV-infected group than in the control group, while the number of exosomes with particle sizes ranging from 70 to 110 nm was lower in the PEDV-infected group. The infection group was significantly smaller than the control group. The average exosome particle size in the PEDV-infected group was higher than in the control group. Immunoblotting analysis confirmed that the exosomes of the PEDV-infected group and the control group expressed exosome marker proteins TSG101, HSP70, β-actin, and CD9, indicating that the purity of exosome secretion *in vitro* met the standard for further experiments (Figure 2C).

### 3.3 Cytotoxicicity evaluation

**Figure 3:**
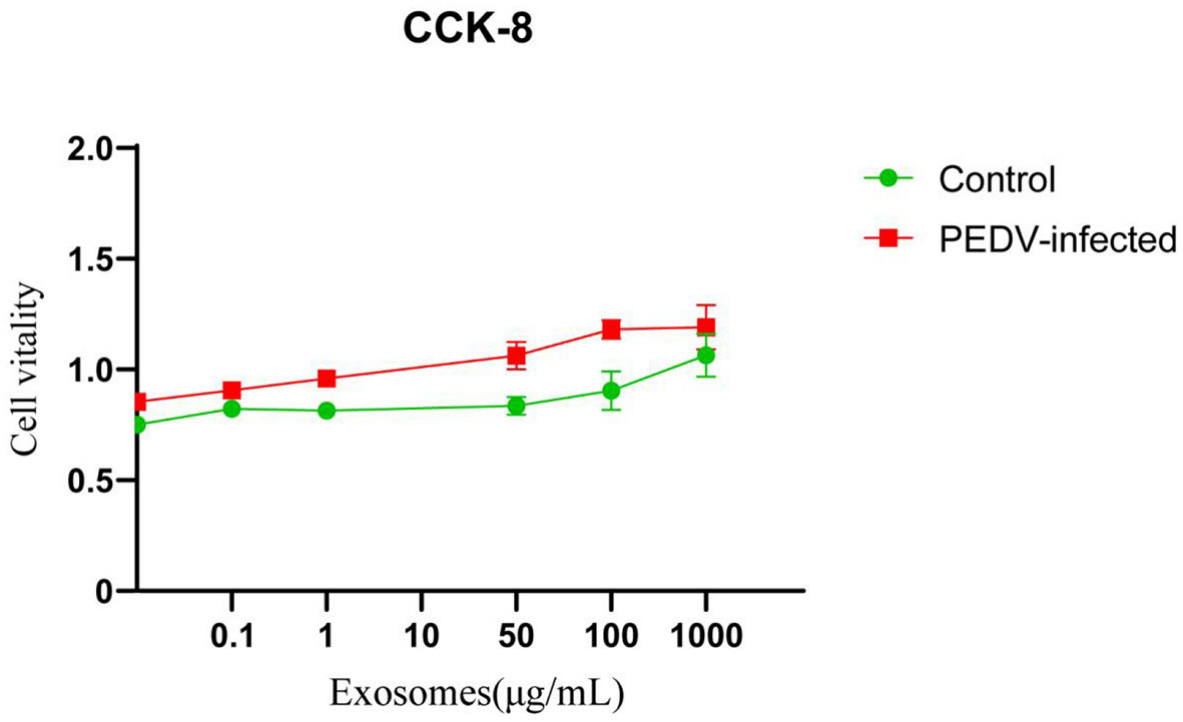
The cytotoxicity of exosomes in Vero cells. Three independent experiments were carried out.

As shown in Figure 3, the results of CCK8 assay showed that no apparent cytotoxicity of exosomes isolated from healthy and PEDV-infected intestines were observed on Vero cells at a concentration ranging from 0 to 1,000 μg/mL.

### 3.4 Analysis of small RNA sequencing library data

After image recognition and base identification, the quality of the Illumina SE50 sequencer raw reads was evaluated. A Q30 score above 85% indicated satisfactory sequencing quality. As shown in Table 3, the Q30 score for the PEDV-infected group was 91.92% and for the control group was 92.01%, indicating that the sequencing data were of good quality and suitable for further analysis. The miRNA libraries were constructed and sequenced from exosomes of PEDV-infected and healthy piglets' small intestinal tissue to investigate the impact of PEDV infection on exosome miRNAs. The raw data from infected tissues yielded 32,312,714 original sequences, and 33,991,672 were obtained from healthy tissues. After removing low-quality labels, adapter sequences, and short reads <15 nt, clean reads were identified in 31,285,653 (the PEDV-infected group) and 32,825,678 (the control group) sequences. The Q30 results for both groups demonstrated that the sequencing data were of high quality and suitable for further analysis.

**Table 3:** Sequencing library raw data.

**Group**	**Raw reads**	**Clean reads**	**Q30**	**Mapped reads**
PEDV-infected	32,312,714	31,285,653	97.25	24,724,365
Control	33,991,672	32,825,678	96.5	23,292,587

### 3.5 Screening of differential miRNAs

**Figure 4:**
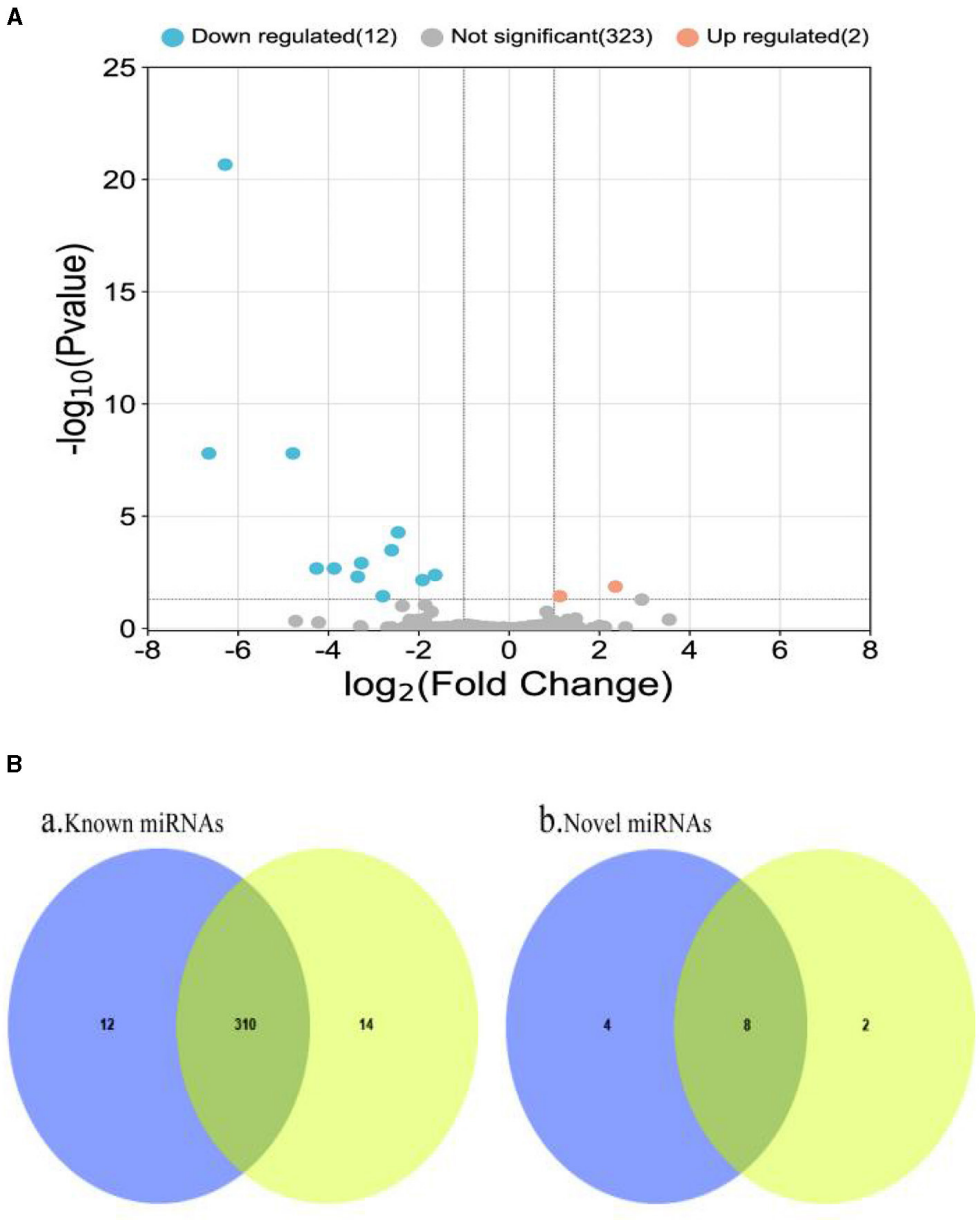
**(A)** Volcano plot of differentially expressed miRNAs. Two miRNAs (ssc-miR-132 and ssc-miR-181d-5p) were up-regulated in small intestinal tissue of healthy piglets, while the remaining 12 were down-regulated. **(B)** Venn diagram of miRNAs shared and unique between groups. It depicts a Venn diagram of the number of common and unique miRNAs found in the PEDV-infected and control groups. Each number denotes the number of miRNAs observed in each group. The number in the overlap area represents the miRNAs shared by the two comparison groups. Each non-overlapping region's number represents the unique miRNA in each group (*P* < 0.05). It can be seen that there were 336 known miRNAs in both the PEDV-infected and control groups, with the infected group having fewer miRNAs than the control group. There were 14 new miRNAs, with the infected group having more than the control group. Blue: miRNAs of PEDV-infected intestinal exosomes. Yellow: miRNAs of healthy intestinal exosomes.

The RPM method was utilized to compare and standardize the expression levels of mature miRNAs. The number of reads for each miRNA was counted and compared to the original expression of the miRNA. The *P*-value and fold changes in exosome samples from small intestinal tissues of pigs infected with PEDV and controls were calculated. Differentially expressed miRNAs were identified based on the screening criteria of fold change ≥2 and *P* ≤ 0.05. The fold change and *P* ≤ 0.05 were used as the differential miRNA screening thresholds. Volcano plots and Venn diagrams were plotted for the data results to visualize the differentially expressed exosomal miRNAs. The Volcano plots displayed the distribution of differentially expressed miRNAs in the PEDV-infected and control groups. The Venn diagram showed the number of differentially expressed miRNAs in both groups. The volcano plots and Venn diagrams are presented in Figures 4A, B, respectively.

### 3.6 Cluster analysis of different miRNAs

**Figure 5:**
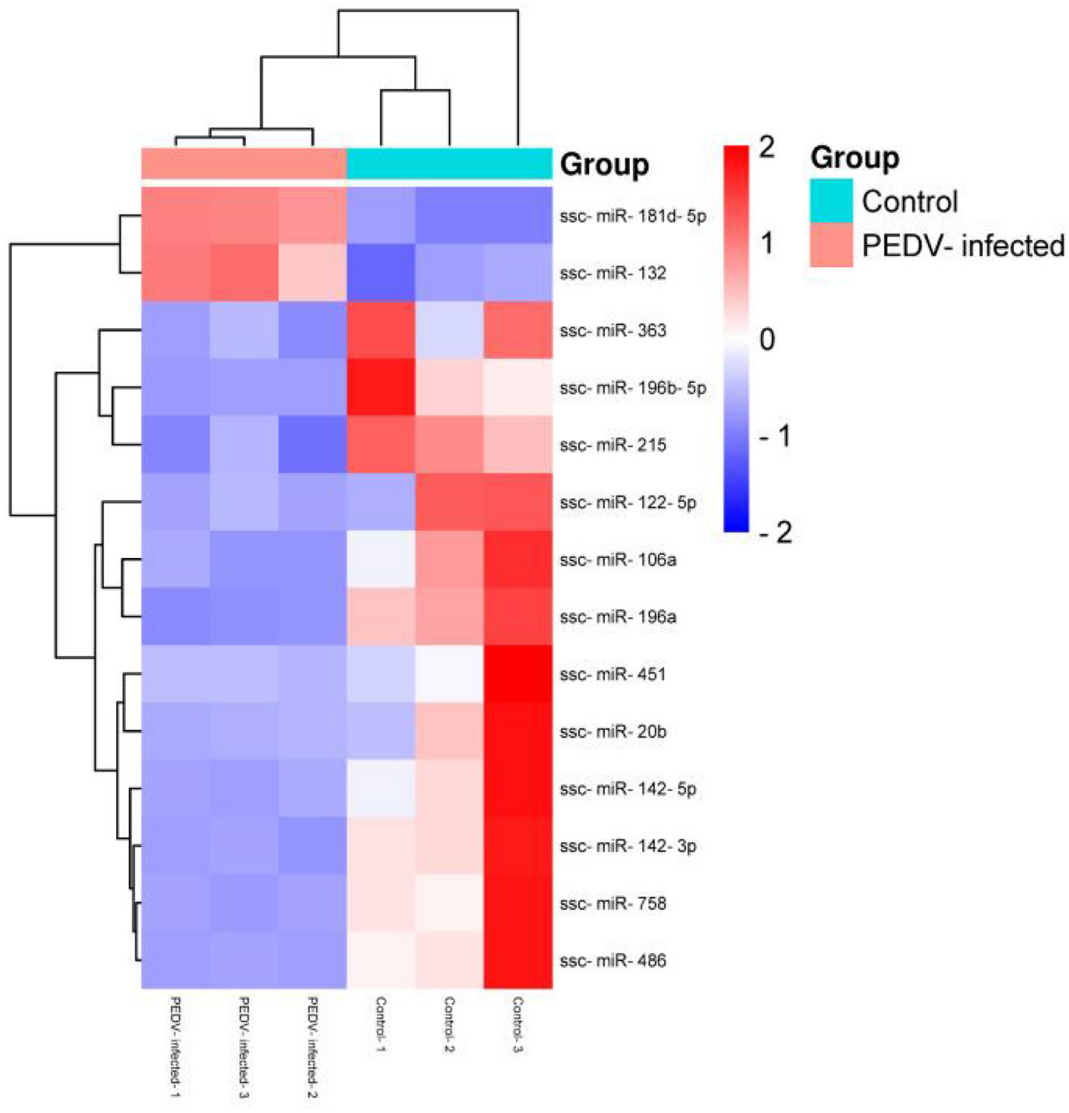
Cluster analysis of differential miRNA expression.

The R program was used to stratify cluster known miRNAs in the PEDV-infected and control groups to identify abnormal expression of exosome miRNAs in the small intestinal tissues of piglets in the PEDV-infected and healthy control groups. The upper part was labeled in red, while the lower part was labeled in blue. Figure 5 shows that 14 exosome miRNAs were differentially expressed between the two groups, with two being up-regulated (ssc-miR-181d-5p and ssc-miR-132) and the others being down-regulated (Table 4).

**Table 4:** Exosome miRNAs of RT-qPCR verification.

**miRNA**	**Reads in PEDV**	**Reads in control**	***P*-value**	**Fold change**	**Related**
ssc-miR-122-5p	213	3,109	5.65 × 10^−5^	0.068510775	Down
ssc-miR-106a	1	25	5.51 × 10^−5^	0.04	Down
ssc-miR-132	49	8	5.45 × 10^−4^	6.125	Up
ssc-miR-142-3p	39	214	7 × 10^−7^	0.182242991	Down
ssc-miR-142-5p	101	381	2.57 × 10^−4^	0.265091864	Down
ssc-miR-181d-5p	255	116	1.59 × 10^−3^	2.198275862	Up
ssc-miR-196a	2	67	1.33 × 10^−10^	0.029850746	Down
ssc-miR-196b-5p	2	73	1.58 × 10^−10^	0.273972603	Down
ssc-miR-20b	4	38	1.66 × 10^−4^	0.105263158	Down
ssc-miR-215	2,146	13,014	5.44 × 10^−6^	0.161899339	Down
ssc-miR-363	269	837	1.25 × 10^−4^	0.321385902	Down
ssc-miR-451	984	9,518	2.45 × 10^−5^	0.103383064	Down
ssc-miR-486	16	1,204	7.26 × 10^−24^	0.013289037	Down
ssc-miR-758	2	15	1.76 × 10^−3^	0.133333333	Down

### 3.7 Validation of miRNA results by RT-qPCR

**Figure 6:**
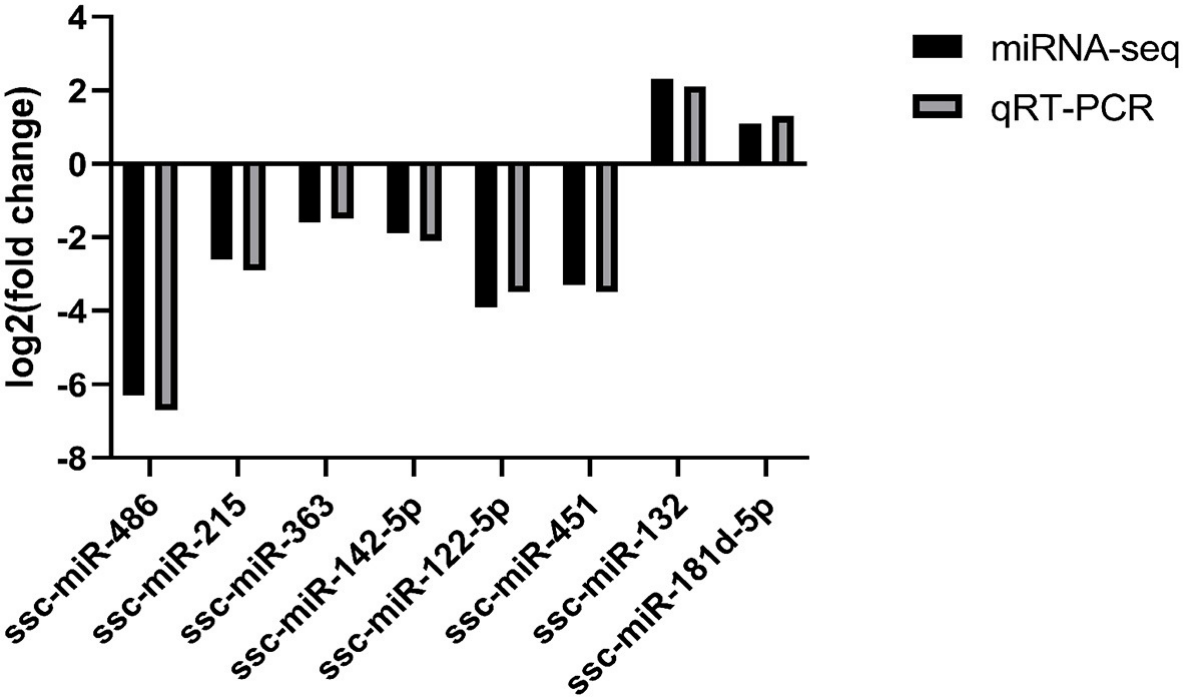
Fluorescence quantitative PCR validation.

The sequencing data were subjected to bioinformatics analysis, and 14 differentially expressed miRNAs were identified using the RT-qPCR method. Eight of these miRNAs were randomly selected for verification by fluorescence quantitative PCR. Two of the selected miRNAs, ssc-miR-132 and ssc-miR-181d-5p, were found to be up-regulated (Figure 6). Additionally, six miRNAs, including ssc-miR-486, ssc-miR-215, ssc-miR-363, ssc-miR-142-5p, ssc-miR-122-5p, and ssc-miR-451, were down-regulated. The expression trends of these differentially expressed genes were consistent with the results of RNA-seq sequencing, validating the accuracy of the sequence analysis.

### 3.8 Analysis of GO enrichment and pathway prediction of differential miRNAs

**Figure 7:**
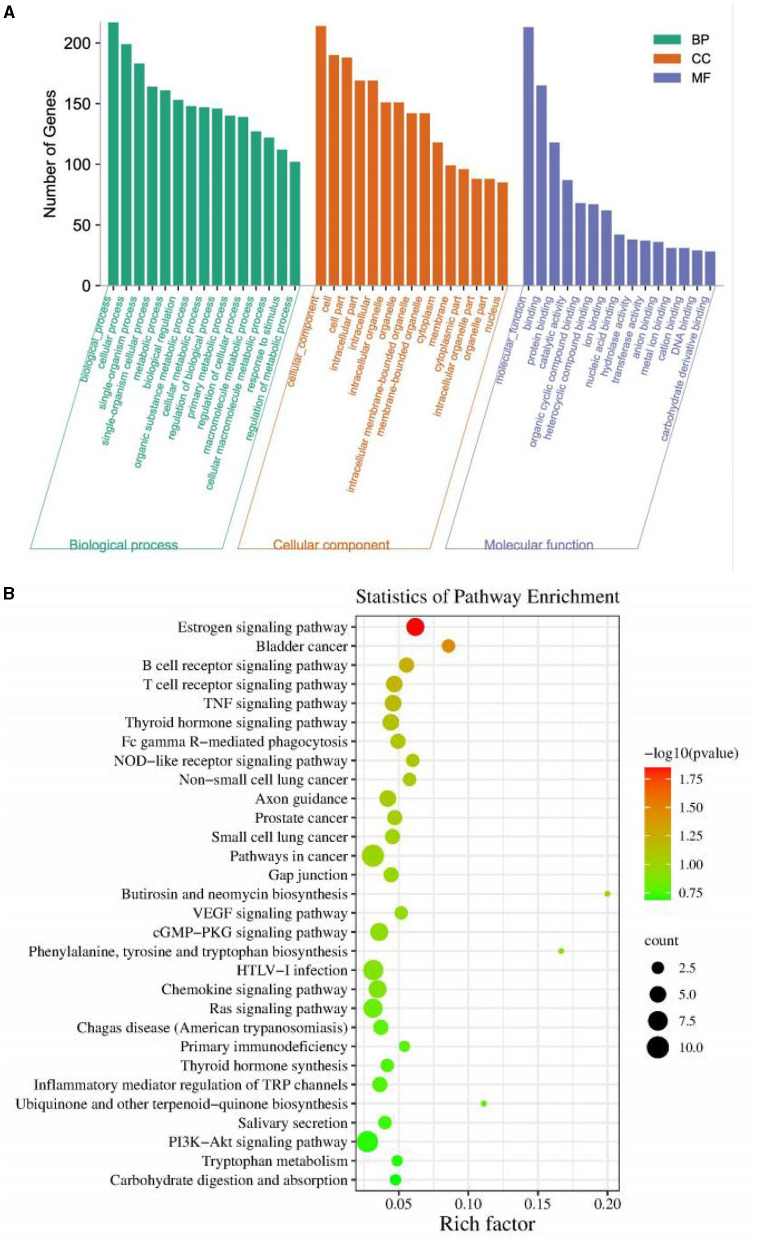
**(A)** Gene Ontology classification of target genes. **(B)** KEGG enrichment pathway of target genes.

GO enrichment analysis was carried out on all identified differential miRNAs, and the annotation function categories were classified into three groups: biological process (BP), cell component (CC), and molecular function (MF). The top enriched categories in the BP group were biochemical processes, cellular processes, and single-organism processes (Figure 7A). In the CC group, the top enriched categories were cellular component, cell part, and intracellular region (Figure 7B). In the MF category, protein binding, catalytic activity, and organic cyclic compound binding were the most enriched (Figure 7B). KEGG signaling pathway analysis was performed on the target genes predicted by differential miRNAs, and the top 30 pathways with the highest enrichment were analyzed (Figure 7B). The estrogen signaling pathway, bladder cancer, B cell receptor signaling pathway, T cell receptor signaling pathway, TNF signaling pathway, thyroid hormone signaling pathway, FC-γR mediated phagocytosis, NOD-like receptor signaling pathway, non-small cell lung cancer, axon guidance, prostate cancer, small cell lung cancer, pathway in cancer, gap junction, Butirosin and neomycin biosynthesis, VEGF signaling pathway, cGMP-PKG signaling pathway, phenylalanine, tyrosine and tryptophan biosynthesis, HTLV-I infection, chemokine signaling pathway, Ras signaling pathway, Chagas disease (American trypanosomiasis), primary immunodeficiency, thyroid hormone synthesis, inflammatory mediators regulation of TRP channels, ubiquinone and other terpenoid-quinones biosynthesis, salivary secretion, PI3K-Akt signaling pathway, tryptophan metabolism, and carbohydrate digestion and absorption were included. These results suggest that PEDV infection significantly impacts various signaling pathways in the host.

## 4 Discussion

Porcine epidemic diarrhea (PED) is a highly contagious and acute intestinal disease in piglets caused by PED virus (PEDV) infection (Shen et al., 2020). The infection alters miRNA expression in host cells, creating a complex network of interactions between the virus and the host (Zheng et al., 2018; Zhang X. et al., 2021). PEDV infection has been shown to significantly affect the intestinal microflora of newborn piglets, decreasing the number of beneficial bacteria such as Bacteroides and increasing the levels of harmful bacteria, thereby disrupting the balance of the intestinal flora (Johnstone et al., 1987). Some studies have shown that it was observed that after PEDV infects piglets, the virus infects and replicates in the piglets' intestines, leading to atrophy of intestinal villi, swelling of superficial villous enterocytes, vacuolization in the cytoplasm, and enlarged nuclei in some intestinal cells. Additionally, there was necrosis and detachment of mucosal epithelial and lamina propria cells, as well as dilated and congested blood vessels. Similarly, the large intestinal glands in the cecum were disorganized, with cell disintegration and detachment, and a small number of lymphocytes were present in the submucosal layer (Li et al., 2022). These changes cause significant damage to the piglets' intestines, leading to severe watery diarrhea and malabsorption. The resulting allergic reactions and co-infections further exacerbate the disease. Therefore, understanding the mechanisms underlying PEDV infection and its impact on the host is crucial for developing effective prevention and control strategies for PED.

The study of exosomes began in the 1980s, and it was discovered that the quality of plasma membrane protein affects the production of exosomes (Trams et al., 1981; Pan et al., 1985). The clearance mechanism of the transferrin receptor (TFR) on the plasma membrane of mature reticular cells was also investigated, and it was found that selective endocytosis of the transferrin receptor occurred on the plasma membrane, which ultimately leads to the formation of multivesicular bodies (MBV) (Pan et al., 1985; Gruenberg, 1986). Furthermore, exosomes are also produced in the plasma membrane through endocytosis (Conner and Schmid, 2003). Exosomes have a broad range of biological functions, including intercellular signaling and involvement in various biological processes (Zhang et al., 2015). High-throughput sequencing technology has been used to reveal the differential expression of miRNA, which has helped to clarify the mechanism of interaction between PEDV and the host (Li et al., 2018). However, whether exosome miRNAs influence PEDV replication by regulating the host immune response and the target virus remains unclear. Further studies are needed to explore the potential role of exosome miRNAs in modulating PEDV infection and replication.

Exosomes are small, membrane-bound vesicles that measure 30–150 nm in diameter and are released by various cells (Raposo and Stoorvogel, 2013; Colombo et al., 2014; Kalluri and LeBleu, 2020). They express specific biomarkers such as CD63, CD9, and CD81 on their surface and can be found in multiple body fluids (Raposo and Stoorvogel, 2013; Colombo et al., 2014; Kalluri and LeBleu, 2020). Exosomes are important mediators of intercellular communication and can transport different biologically active molecules, including proteins, mRNA, and miRNA, to target cells (Raposo and Stoorvogel, 2013; Colombo et al., 2014; Kalluri and LeBleu, 2020). While exosomes have been shown to play a role in various viral infections, their relationship with PEDV infection is not well understood (Li et al., 2018). However, exosomes have been shown to have antiviral effects in some cases. For example, exosomes released by human T cells infected with HTLV-1 can activate the immune response and stimulate the production of inflammatory cytokines (Gaudin et al., 2011; Igawa et al., 2011; Zhang et al., 2011; Li et al., 2013; Zhou et al., 2018). Similarly, exosomes released during dengue virus infection have been shown to have various antiviral effects (Buddingh et al., 2015). On the other hand, exosomes can also enhance viral replication and infection in some cases. For instance, the assembly and release pathways of exosomes and influenza A virus (IAV) and respiratory synthetic virus (RSV) overlap significantly, leading to increased viral replication and infection (Zhou et al., 2018). Our study found that healthy small intestinal exosomes could inhibit PEDV activity, suggesting that exosomes may competitively bind to specific virus-specific recognition receptors on the surface of host cells, preventing the virus from entering the cell to replicate and achieve antiviral effects. The surface of PEDV is composed of several proteins, including the nucleocapsid (N) protein, which is mainly responsible for viral particle assembly, host stress, and immune response (Zhang Y. et al., 2016). Further studies are needed to better understand the relationship between exosomes and PEDV infection and their potential therapeutic applications.

The yield and purity of target exosomes are influenced by various factors during centrifugation, including time, force, rotor type, and more (Johnstone, 1992). Although this method prevents cross-contamination without labeling, it is challenging due to its lengthy execution, high cost, structural damage, and lipoprotein co-separation. Density gradient centrifugation is used to purify exosomes and is often combined with ultracentrifugation to enhance exosome purity. However, due to their similar size and density, exosomes and retroviruses cannot be separated using sucrose density gradients, and their deposition rates in the iodovenol gradient are significantly different, allowing for the successful separation of exosomes from PEDV-infected intestinal tissues and the extraction of high-purity exosomes. Density gradient centrifugation is advantageous for exosome purity, but the high viscosity of sucrose solution decreases the rate of exosome precipitation, leading to a longer time. To address this, we used a combination of density gradient centrifugation and ultracentrifugation to separate exosomes from piglet intestinal tissues, which were then identified using NTA, western blotting, and exosome-labeled proteins TSG101, HSP70, β-actin, and CD9 for quantitative analysis. The results indicated that we successfully obtained exosomes from piglet intestinal tissue with a purity that met the standard, providing a foundation for future experimental studies.

The intestinal microbiota serves as the digestive system's microbial barrier, maintaining a balanced ecosystem under typical circumstances that resist harmful bacteria adhesion, colonization, and invasion. However, disrupting the equilibrium of intestinal microbes may result in various disorders and negatively impact the body's health. Exosomes function as endogenous regulatory substances that can influence the composition and growth of intestinal flora. Certain research has indicated that exosomes can interact with intestinal flora and the body, altering the composition and abundance of intestinal flora. Teng et al. discovered that intestinal microbiota could take up plant-derived exocrine nanoparticles containing RNA that modifies microbial composition and the host. Yu et al. found that combining bovine milk exosomes with Escherichia coli and lactic acid bacteria significantly increased the production rate of Escherichia coli and lactic acid bacteria. Exosomes contain miRNA, which plays crucial regulatory roles in various biological processes, primarily regulating genes at the post-transcriptional level (Teng et al., 2018). Exosomes can specifically choose miRNAs during packaging, directly targeting viral genomic RNA and inhibiting viral replication in host-virus interactions, thereby playing a vital regulatory role. Understanding the role of miRNA in the small intestine tissues of exosomes after PEDV infection is essential to elucidate miRNA's role in intracellular communication and antiviral response induction.

After evaluating the cytotoxicity of extracellular vesicles, it was found that extracellular vesicles isolated from healthy and PEDV infected intestines ranged from 0 to 1,000 μ. No significant cytotoxicity was observed on Vero cells within the concentration range of g/ml. In this study, miRNAs were extracted and sequenced from the intestinal exosomes of piglets, and 350 miRNAs were identified, including 336 known and 14 differentially expressed miRNAs. These miRNAs may be involved in the interaction of exosomes with PEDV in porcine intestinal tissues, which may in turn regulate virus replication. As important carriers of intercellular communication and transfer of genetic material, exosomes carry miRNAs involved in the regulation of various biological processes. Although a variety of miRNAs are known to be involved in the process of viral infection, there are fewer reports on the involvement of specific miRNAs in porcine epidemic diarrhea virus (PEDV) infection. Among these, 14 unique miRNAs were clustered and analyzed, and 8 abnormally expressed miRNAs were selected for RT-qPCR validation. The results revealed that two genes (ssc-miR-181d-5d and ssc-miR-132) were up-regulated, while the remaining were down-regulated, and among the 12 significantly down-regulated miRNAs, the expression of ssc-miR-486, ssc-miR-215, and ssc-miR-451 was higher, and there might be novel miRNAs regulating PEDV replication, confirming their transcriptional accuracy. The specific mechanism of action still needs to be further explored.

GO and KEGG pathway analyses showed that the differentially expressed miRNA target genes were mainly involved in cellular process signaling pathways, including B-cell receptor signaling pathway, Ras signaling pathway, cGMP-PKG signaling pathway, and PI3K-Akt signaling pathway. During hepatitis E virus (HEV) replication, estrogen signaling in association with STAT3, regulates interferon expression by modulating hepatocyte function and host innate immune response (Sooryanarain et al., 2022). In related studies on signaling and antivirality, carbon monoxide was shown to inhibit PRRSV replication through activation of the cGMP/PKG signaling pathway; carbon monoxide significantly inhibited PRRSV-induced NF-κB activation, decreased PRRSV-induced anti-inflammatory cytokine mRNAs, and exhibited antiviral effects (Zhang A. et al., 2016). Therefore, the signaling pathways enriched for the target genes of differentially expressed miRNAs analyzed in our study may affect the replication mechanism of PEDV, which requires further investigation.

These findings suggest that abnormally expressed exosomal miRNAs were detected in the intestinal exosomes of PEDV-infected piglets, indicating the presence of the PEDV virus in the small intestinal tissues, which underwent infection and replication, resulting in severe damage to the piglet intestine. Furthermore, the cleanest reads in PEDV-infected and PEDV-uninfected piglets were 21–24 nt in length, with the highest RNA content being 22 nt. The pathological changes in the intestinal tissues and the abnormally expressed exosomal miRNAs detected in the intestinal exosomes of PEDV-infected piglets suggest that the PEDV virus infected and replicated in the piglet's small intestinal tissues, leading to severe damage to the intestine. The findings of this study could provide valuable insights into the mechanisms underlying PEDV infection and pathogenesis.

The results of this study align with the typical size of miRNA derived from Dliller, indicating an abundance of miRNA sequences in the library (Zhang L. et al., 2021). Specifically, there were abnormally regulated miRNAs found in the exosomes of porcine small intestine tissue following PEDV infection, suggesting a potential role in the mechanism of PEDV-host interaction. These abnormally regulated miRNAs may serve as important indicators; however, the specific mechanism of their involvement requires further investigation. Therefore, these findings provide a foundation for understanding the PEDV-host interaction and highlight the potential significance of miRNA in this process.

## 5 Conclusion

This study successfully isolated high-purity, PEDV-free porcine small intestinal tissue exosomes and PEDV-infected exosomes. Through this, differentially expressed miRNAs were detected in piglet intestinal tissue exosomes. Specifically, ~350 miRNAs were identified in the secretions of PEDV-infected porcine small intestinal tissues, including 14 newly identified miRNAs. Among these, 14 miRNAs were differentially expressed in the small intestinal tissue secretions of PEDV-infected pigs, two of which were up-regulated while the rest were down-regulated. Similarly, 14 miRNAs were differentially expressed in PEDV-infected pig small intestinal secretions, two of which were up-regulated while the rest were down-regulated. This study has identified aberrantly regulated miRNAs in the small intestinal secretions of pigs infected with PEDV. These findings suggest that future observation and analysis of these aberrantly regulated miRNAs may aid in understanding the interaction mechanism between PEDV and the host. Overall, this research provides a foundation for further investigation into the role of miRNA in PEDV infection and pathogenesis.

## Data availability statement

The data presented in the study are deposited in the BioProject database, accession number PRJNA1105240.

## Ethics statement

The animal studies were approved by College of Life Science and Engineering, Foshan University. The studies were conducted in accordance with the local legislation and institutional requirements. Written informed consent was obtained from the owners for the participation of their animals in this study.

## Author contributions

JW: Writing – original draft, Writing – review & editing. LS: Writing – original draft, Writing – review & editing. GM: Writing – review & editing. YW: Writing – review & editing. YL: Writing – review & editing. SE-A: Writing – review & editing. RA: Writing – review & editing. ZL: Data curation, Formal analysis, Funding acquisition, Writing – review & editing.
